# A long-term survivor of clear cell sarcoma-like tumor of the gastrointestinal tract with liver metastasis: a case report

**DOI:** 10.1186/s40792-020-01028-z

**Published:** 2020-10-06

**Authors:** Takuhisa Okada, Yasumitsu Hirano, Shintaro Ishikawa, Hiroka Kondo, Toshimasa Ishii, Shigeki Yamaguchi

**Affiliations:** 1 Department of Gastroenterological Surgery, Saitama Medical University International Medical Center, 1397-1, Yamane, Hidaka-City, Saitama-Pref 350-1298 Japan; 2 Department of General Surgical Science, Gunma University Graduate School of Medicine, 3-39-22 Showa-Machi, Maebashi-City, Gunma-Pref 371-8511 Japan

**Keywords:** Clear cell sarcoma-like tumor of the gastrointestinal tract, Liver metastasis, Long-term survival

## Abstract

**Background:**

Clear cell sarcoma-like tumor of the gastrointestinal tract (CCSLTGT) is extremely rare. It is a mesenchymal neoplasm that usually forms in the small intestine of adolescents and young adults, is prone to local recurrence and metastasis, and has a high mortality rate. We report a patient with CCSLTGT with lymph node- and liver metastases, who continues to survive 6 years after initial surgical resection.

**Case presentation:**

A 38-year-old woman presented with lightheadedness. Laboratory analysis revealed anemia (hemoglobin, 6.7 g/dL), and enhanced computed tomography (CT) demonstrated a mass in the small intestine, about 6 cm in diameter, with swelling of 2 regional lymph nodes. Double-balloon small intestine endoscopic examination revealed a tumor accompanied by an ulcer; the biopsy findings suggested a primary cancer of the small intestine. She was admitted, and we then performed a laparotomy for partial resection of the small intestine with lymph node dissection. Pathologic examination revealed CCSLTGT with regional lymph node metastases. About 3 years later, follow-up CT revealed a single liver metastasis. Consequently, she underwent a laparoscopic partial liver resection. Histopathologic examination confirmed that the liver metastasis was consistent with CCSLTGT. It has now been 3 years without a recurrence.

**Conclusion:**

Repeated radical surgical resection with close follow-up may be the only way to achieve long-term survival in patients with CCLSTGT.

## Introduction

Clear cell sarcoma-like tumor of the gastrointestinal tract (CCSLTGT) is a very rare malignant tumor of mesenchymal origin. Histopathologically, CCSLTGT has characteristic spindle-shaped tumor cells that proliferate in sheets, and osteoclast-like multinucleated giant cells are seen [1, 2]. On immunohistologic staining, S-100 protein and SOX10 are positive but HMB-45 is negative, a finding that distinguishes CCSLTGT from malignant melanoma and soft-tissue clear cell sarcoma (CCS). As with CCS, Ewing sarcoma breakpoint region 1 (EWSR1) gene fusion is noted [3, 4]. Because CCSLTGT is a highly malignant tumor, it is important to distinguish it from other mesenchymal neoplasms, such as gastrointestinal stromal tumors (GIST). Local recurrence is common with CCSLTGT, as are lymph node metastases and distant metastases to the liver and lung. These metastases occur rapidly and usually result in death [2]. We report herein a patient with liver metastasis after resection of CCSLTGT of the small intestine. She is alive, without further recurrence, 6 years after her initial surgery.

## Case report

**Fig. 1:**
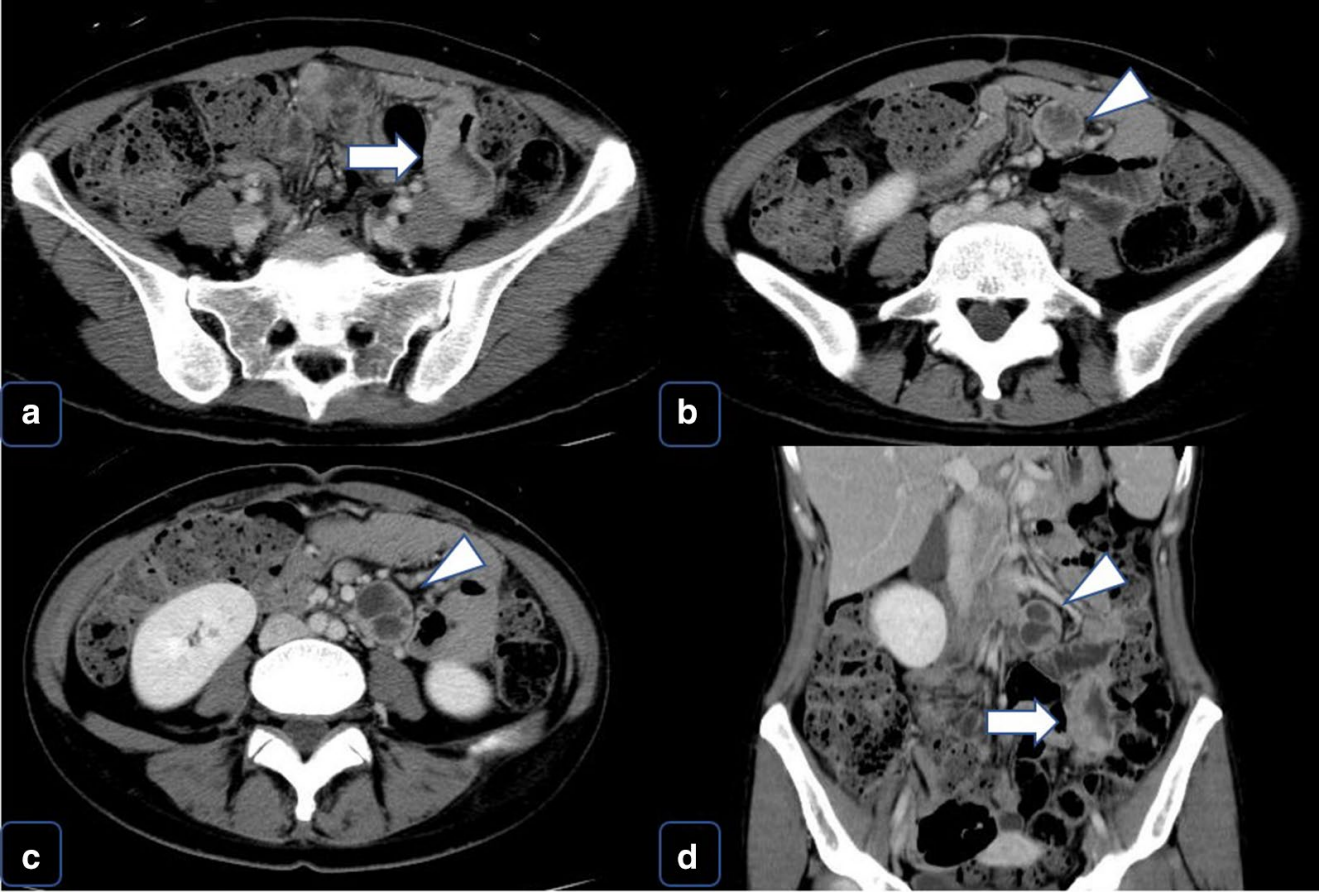
CT findings. **a** Horizontal view. Primary tumor (the white arrow). **b**, **c** Horizontal view. Lymph nodule (the arrowhead). **d** Sagittal view. Primary tumor (the white arrow) and lymph nodule (the arrowhead)

**Fig. 2:**
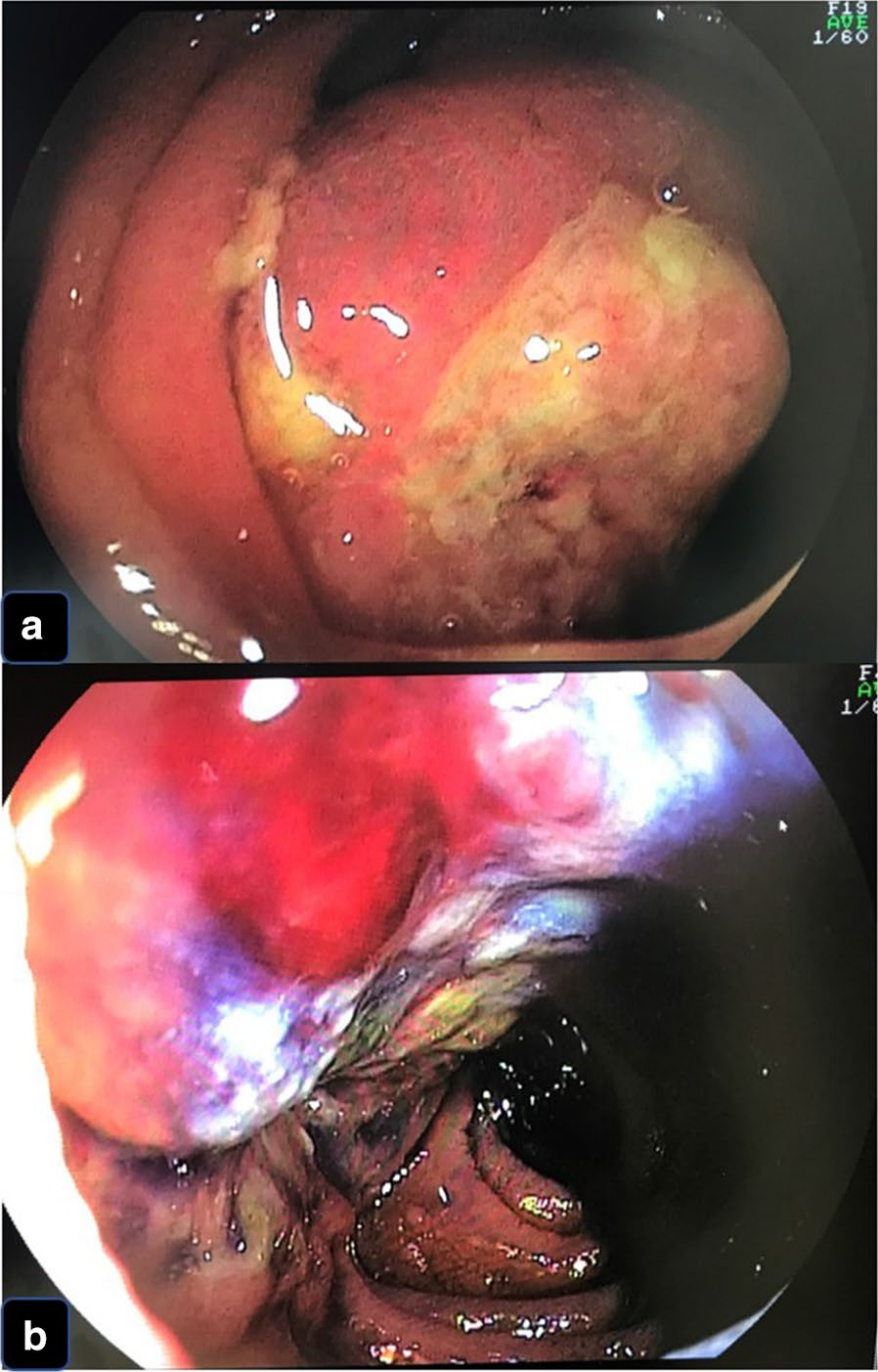
Findings of double-balloon endoscopy. **a**, **b** Primary tumor looked like submucosal tumor accompanied by an ulcer

**Fig. 3:**
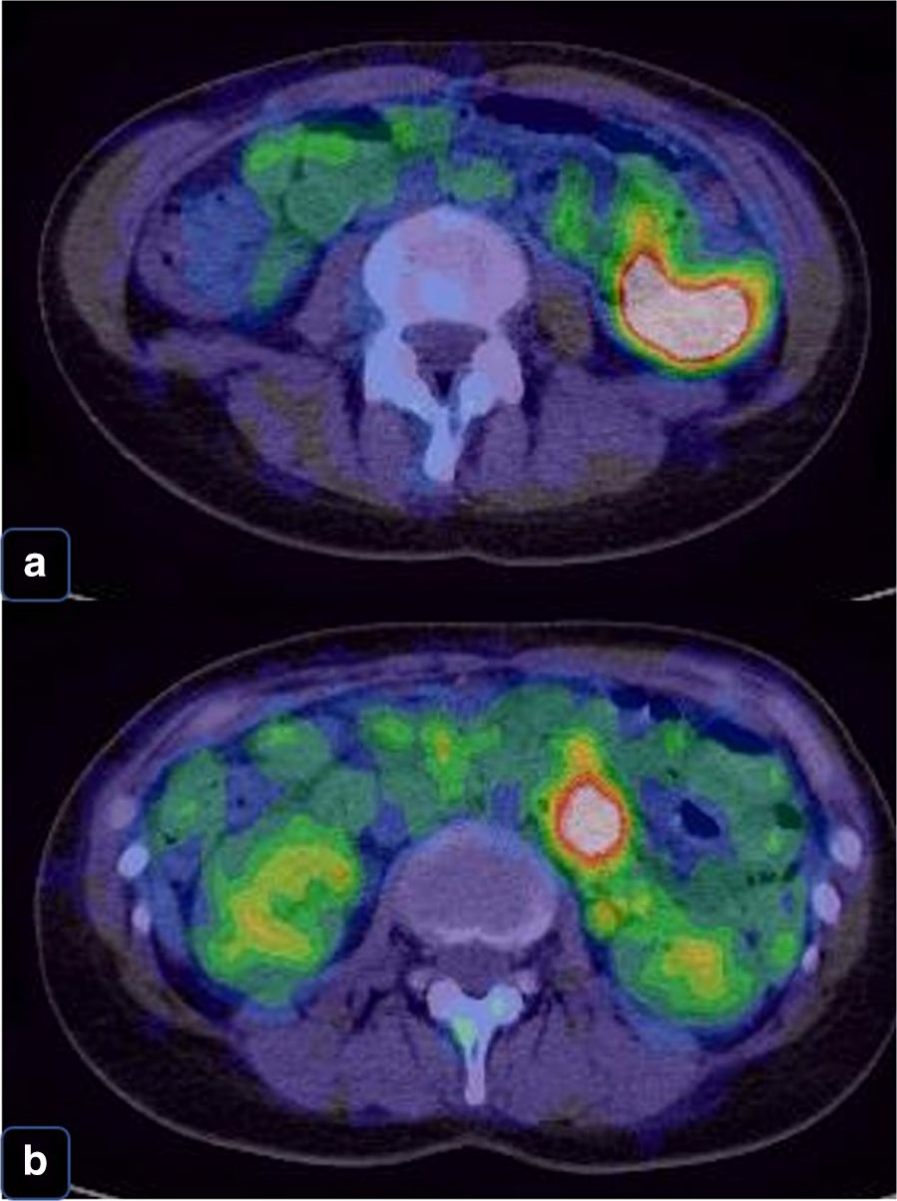
PET–CT confirmed an abnormal accumulation of F-18 fluorodeoxyglucose in the tumor and lymph nodes. **a** Primary tumor’s accumulation. **b** Lymph nodule’s accumulation

**Fig. 4:**
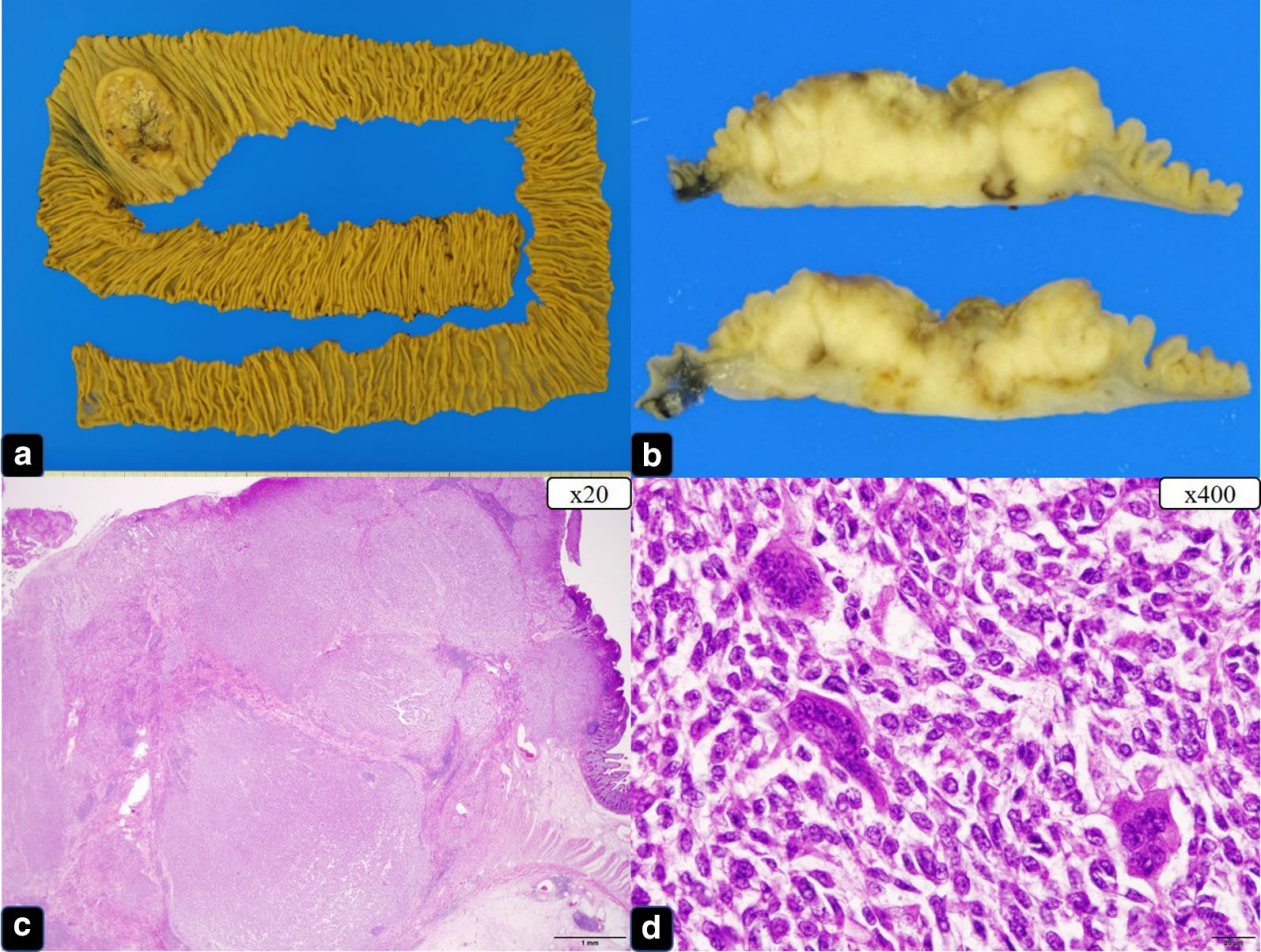
The tumor was located in the jejunum and was an ulcerative lesion. **a** Resected specimen (jejunum). **b** Cut surface, white solid tumor with clear boundary. **c** HE stain, low-power view. **d** HE stain, high-power view

**Fig. 5:**
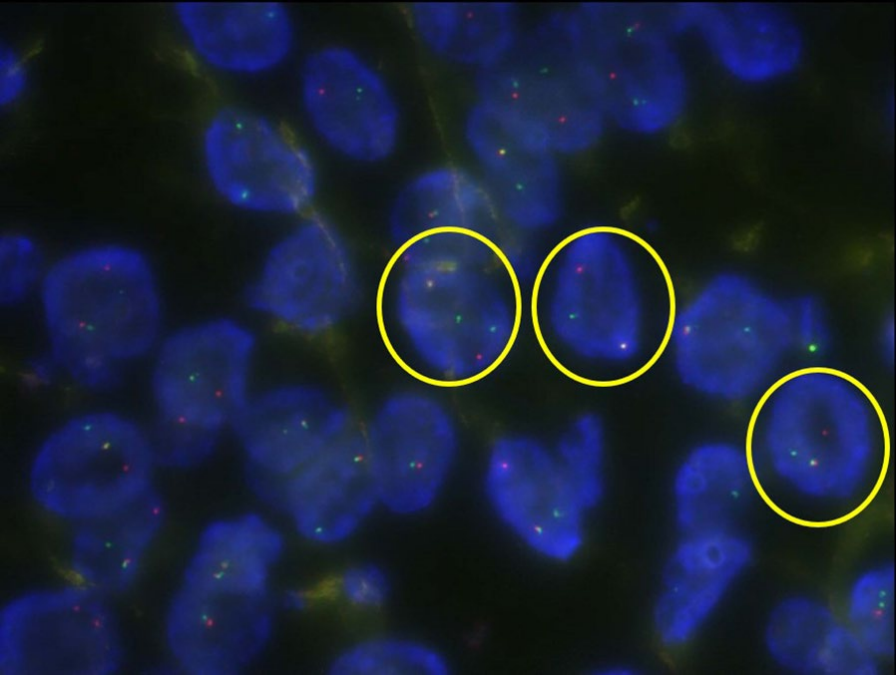
EWSR1-FISH positive lesion (yellow circle)

A 38-year-old Japanese woman presented with lightheadedness. Her medical history was significant for malignant lymphoma at the age of 18 years. Laboratory analysis revealed anemia (hemoglobin, 6.7 g/dL), and enhanced computed tomography (CT) revealed a mass in the small intestine measuring about 6 cm in diameter, with swelling of 2 regional lymph nodes (Fig. 1). The patient underwent a double-balloon small intestine endoscopy, which revealed a submucosal tumor accompanied by an ulcer (Fig. 2). Examination of the biopsy specimen with immunostaining was negative for malignant lymphoma and GIST, and a very poorly differentiated cancer of the small intestine was suspected. The use of positron emission tomography–CT (PET–CT) confirmed an abnormal accumulation of F-18 fluorodeoxyglucose in the tumor and lymph nodes (Fig. 3). We conferred the diagnosis of a primary malignant tumor of the small intestine with lymph node metastasis. She was admitted and underwent a laparotomy for partial resection of the small intestine with lymph node dissection. The tumor was located in the jejunum and was an ulcerative lesion, measuring 5.8 × 3.9 cm. Gross examination revealed a white, solid tumor with a clear boundary (Fig. 4a, b). The tumor was located between the muscularis mucosa and the muscularis propria of the small intestine (Fig. 4c, d). There was a fibrous septum separating the sheet-like epithelial cells from a network of eosinophilic cells and an oval-shaped bundle of spindle-like tumor cells. The tumor demonstrated a pseudorosette pattern around several blood vessels and displayed a pseudopapillary growth pattern. Throughout the neoplasm, the tumor cells were associated with numerous osteoclast-type giant cells. On immunohistochemical examination (Table 1), the tumor cells stained positive for the anticytokeratin antibodies AE1 and AE3 (on part of the surface layer) and for S-100, and partially positive for the neural cell adhesion molecule (CD56) and synaptophysin. The cells stained negative for anticytokeratin (CAM 5.2), lymphocyte common antigen (CD45), desmin, α-smooth muscle actin (ɑ-SMA), c-kit, CD34, HMB-45, melan-A, chromogranin A, CD1a, and CD21. The osteoclast-type giant cells were positive for CD68. Fluorescence in situ hybridization (FISH) using an EWSR1 probe revealed a split signal in the tumor cells: seemingly an EWSR1 gene fusion (Fig. 5). Taken together, these results yielded a diagnosis of CCSLTGT. Metastases were found in 3 of 20 lymph nodes.

**Table 1:** The list of immunohistochemical examination for our case

Positive	Negative
AE1/AE3 (part of the surface layer)	CAM 5.2, HMB-45, Melan-A
S-100	Desmin, chromogranin
CD 56 (partial)	α-SMA, c-kit
Synaptophysin (partial)	CD1a, CD21, CD34, CD45
CD68 (only osteoclast-type giant cell)

**Fig. 6:**
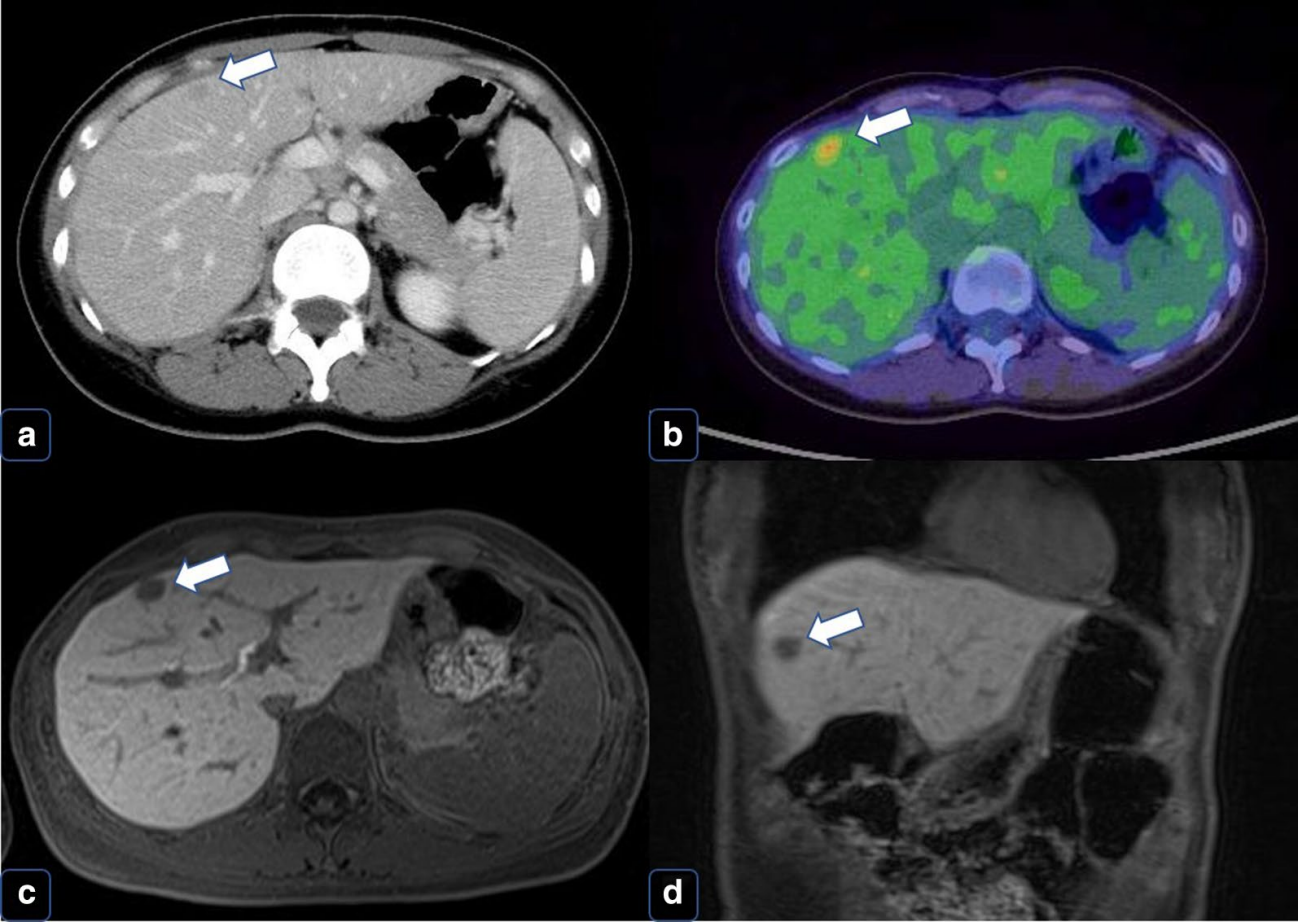
Findings at a single liver metastasis. **a** CT. Horizontal view. Liver metastasis (the white arrow). **b** PET (the white arrow). **c** EOB-MRI. Horizontal view (the white arrow). **d** EOB-MRI. Sagittal view (the white arrow)

**Fig. 7:**
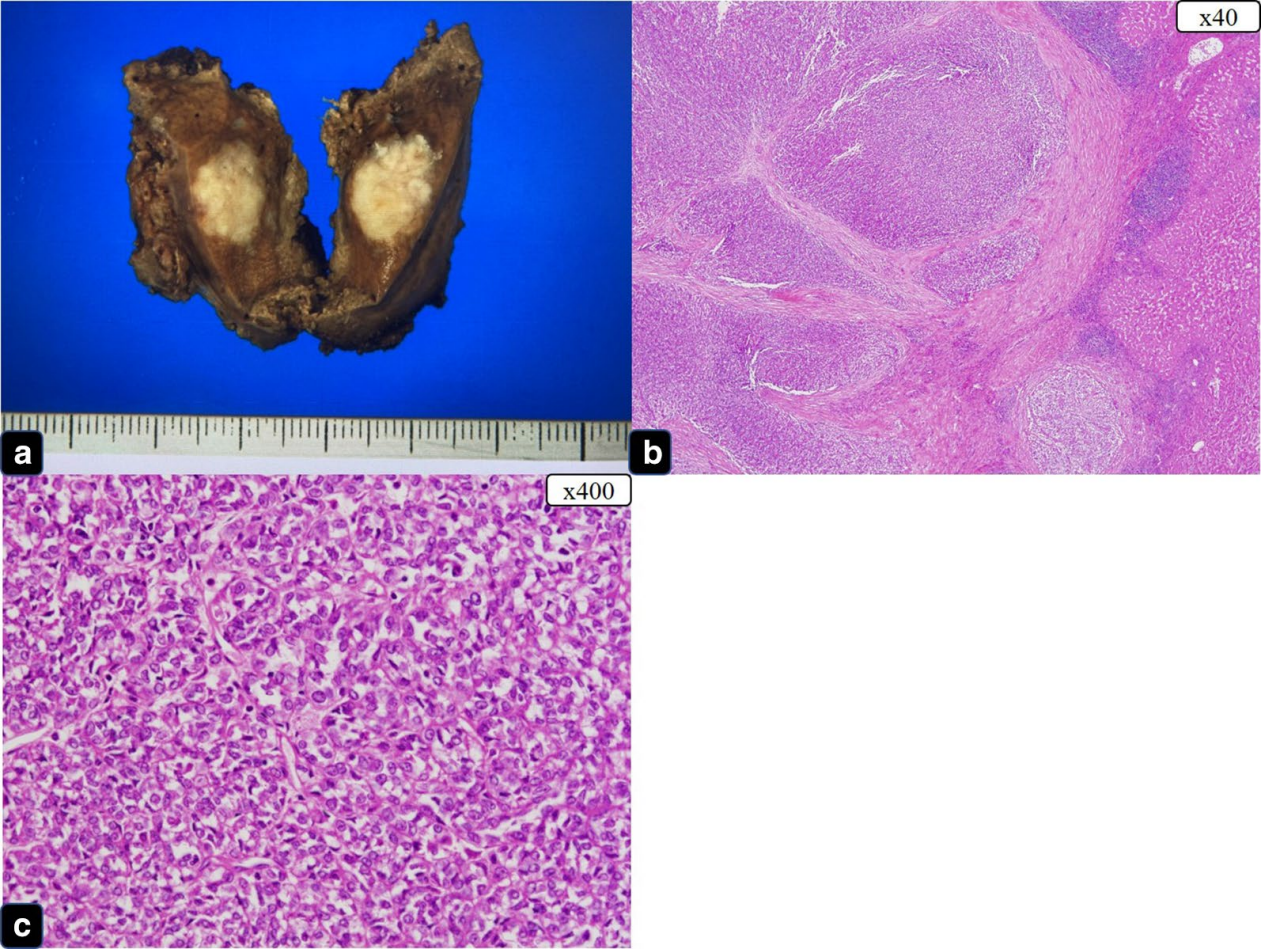
The single metastatic tumor was located in the liver. **a** Resected specimen (liver) and cut surface, white solid tumor with clear boundary. **b** HE stain, low-power view. **c** HE stain, high-power view

About 3 years later, follow-up CT revealed a single liver metastasis (Fig. 6a). The results of gadoxetic acid-enhanced magnetic resonance imaging (EOB-MRI) and PET–CT were also suggestive of liver metastasis (Fig. 6b–d). Consequently, the patient underwent a laparoscopic partial liver resection. Histopathologic examination revealed that the liver metastasis was consistent with CCSLTGT (Fig. 7a–c). It has now been 3 years since this procedure, and follow-up CT every 3 to 6 months has shown no evidence of recurrence.

## Discussion

The entity of CCSLTGT was first described by Zambrano et al. in 2003, when they reported 6 patients with a disease they called “osteoclast-rich tumor of the gastrointestinal tract”, with features resembling those of soft-tissue CCS [1]. This tumor is relatively rare and shows no preference for sex. Many patients are in their 30s and 40s, but there are reports of patients ranging in age from 15 to 85 years [2]. It is most common for CCSLTGT to develop in the small intestine, but it has also been reported in the stomach and colon [2]. Since it is a malignant tumor originating from the interstitium of the wall of the gastrointestinal tract, it must be differentiated from GIST, synovial sarcoma, and malignant peripheral nerve sheath tumors [3]. The etiology of CCSLTGT is unknown, but it has been suggested that radiotherapy may be a precipitating factor in the later development of the disease. Two cases of secondary malignancy after irradiation of neuroblastoma in infancy have been reported [16, 20]. Our case has been treated for malignant lymphoma in the past, but the details of the treatment are unknown. It is not clear whether the treatment was related to the development of CCSLTGT, and there are no reports on the relationship between both malignant lymphoma and CCSLTGT. Scattered osteoclast-like multinucleated giant cells are characteristic of CCSLTGT. Tumor cells stain positive for vimentin, S-100 protein, and SOX10; neuroendocrine markers such as CD56, synaptophysin, and NSE may also be positive. Melanocytic markers such as HMB-45 and melan-A, which are positive in soft-tissue CCS, are negative in CCSLTGT, as are c-kit, CD34, desmin, α-SMA, and keratin. Both our current patient and our previously reported patient [1, 2, 4, 9, 14, 15, 17–19, 21] had osteoclast-like multinucleated giant cells that were positive for CD68, indicating increased responsiveness. Zambrano et al. [1] report a single patient with chromosomal translocation involving EWSR1, similar to that seen with CCS. Cu et al. [4] and Stockman et al. [3] also report patients with CCSLTGT and EWSR gene fusion similar to that seen with CCS. A highly malignant tumor is considered to confer a poor prognosis; many patients die within 36 months, and more than half have local lymph node metastasis at the time of diagnosis. The most common site of distant metastasis is the liver, and curative treatment involves only surgical resection of any metastases. There are 43 reported patients with CCSLTGT of the small intestine to date: 21 men and 22 women, including our previously reported patient (Table 2). The tumor location was the jejunum in 11 patients, the ileum in 24 patients, and was unknown in 8 patients. The sites of metastasis at initial diagnosis include the lymph nodes (*n* = 17), liver (*n* = 6), peritoneum (*n* = 3), and lungs (*n* = 2). Of the 43 patients, curative resection was performed in 30 (69.8%), and among them, recurrence occurred in 18 (60.0%). The mean period between initial surgery and recurrence was 20.0 months. However, 2 recurrences occurred over 5 years after the initial surgery, emphasizing the importance of long-term follow-up. The recurrence sites were the liver (*n* = 11), lymph nodes (*n* = 5), peritoneum (*n* = 4), lung (*n* = 1), ovary (*n* = 1), omentum (*n* = 1), and pleura (*n* = 1). Local recurrence and intra-abdominal recurrence occurred in 1 patient each. Of the 18 patients with recurrence, treatment after recurrence included surgery-alone (*n* = 4), chemotherapy-alone (*n* = 2), surgical resection with chemotherapy (*n* = 1), no treatment (*n* = 1), and unknown details (*n* = 10). All five patients with surgical resection survived within the observation period, among which two patients with curative resection were disease-free and the other three were in the tumor-bearing. Only three patients received chemotherapy. Platinum-based chemotherapy was performed in one patient and details were unknown in two. Two patients with chemotherapy-alone died in 13 and 16 months after recurrence, and their survival times indicated the non-effectiveness of this therapy. Eleven of the reported patients are known to be dead of their disease, but data on mortality are missing for 17 patients. Of the remaining 15 patients, 9 have no evidence of disease, and 6 are alive with disease. Curative surgical resection is the only possible cure treatment for recurrence of CCSLTGT. Chemotherapy has not been reported to be effective, and there are no established chemotherapy regimens until now. In terms of postoperative adjuvant chemotherapy, mainly ifosfamide and doxorubicin were administrated, however, its efficacy has not been proved [28]. Only 2 patients, including the patient in this report, survived for over 5 years without evidence of disease; both of them had tumor recurrence that was treated with surgical resection. Repeated radical surgical resection appears to be the only way to achieve long-term survival in patients with CCSLTGT, a neoplasm that easily metastasizes in a short period. It is important to follow patients with contrast CT every 3 months to ensure that radical resection of any recurrence can occur in a timely fashion.

**Table 2:** Review of reported CCSLGT

Case	Reference	Age	Gender	Primary site	Status at diagnosis	Metastasis	Radial resection	Recurrence (Mo)	Site of recurrence	Therapy after recurrence	Outcome (Mo)
1	1	15	F	Jejunum	Metastatic	Mesenteric LN	No	–	–	–	DOD (16)
2	1	21	F	Ileum	Metastatic	Liver and LN	NR	NR	–	–	DOD (12)
3	1	35	F	Ileum	Metastatic	LN	Yes	Yes (12)	Liver	NR	NR
4	1	37	F	Ileum	NR	NR	NR	NR	–	–	NR
5	1	32	M	Ileum	Metastatic	LN	Yes	NR	–	–	NR
6	5	37	M	Ileum	Localized	–	Yes	Yes (24 and 46)	Liver	Resection	AWD (46)
7	6	57	M	Jejunum	Localized	–	Yes	No	–	–	NED (11)
8	7	35	M	Ileum	Localized	–	Yes	Yes (2 → 7)	Liver → peritoneum	Platinum-based chemotherapy	DOD (15)
9	8	21	F	Ileum	Metastatic	LN	Yes	NR	–	–	NR
10	9	41	M	Jejunum	Localized	–	Yes	Yes (6)	Liver	NR	NR
11	10	85	F	Ileum	Metastatic	LN	Yes	NR	–	–	DOD (1)
12	4	42	F	Ileum	NR	NR	Yes	NR	–	–	NR
13	4	42	F	Ileum	Metastatic	Liver and peritoneal	NR	NR	–	–	NR
14	4	51	F	Ileum	Localized	–	Yes	Yes (60)	Liver and peritoneum	NR	AWD (60)
15	4	18	F	Small intestine	Localized	–	Yes	Yes (NR)	Local	NR	NR
16	11	31	F	Ileum	Metastatic	LN	Yes	NR	–	–	NR
17	12	46	M	Jejunum	Localized	–	Yes	No	–	–	NED (7)
18	12	62	M	Ileum	Metastatic	Mediastinal LN and lung	No	–	–	–	DOD (12)
19	12	60	M	Jejunum	Metastatic	LN	Yes	Yes (12)	Intra-abdominal	Chemotherapy (No details)	DOD (28)
20	13	37	M	Jejunum	Localized	–	Yes	Yes (2)	Peritoneal extension and pleural effusion	NR	NR
21	14	20 s	F	Ileum	Localized	–	Yes	No	–	–	NED (24)
22	15	60	M	Ileum	Metastatic	LN and liver	NR	NR	–	–	NR
23	15	46	M	Jejunum	Metastatic	LN and liver	NR	NR	–	–	NR
24	16	15	M	Ileum	NR	NR	NR	NR	–	–	NR
25	17	16	M	Ileum	Localized	–	Yes	Yes (0.5)	Mesenteric LN, omentum and lung	NR	DOD (1)
26	18	53	F	Ileum	Metastatic	Regional LN	Yes	No	–	–	NED (7)
27	18	26	F	Small intestine	Localized	–	NR	NR	–	–	NR
28	18	66	M	Ileum	Metastatic	Regional LN	Yes	No	–	–	NED (NR)
29	19	69	F	Ileum	Localized	–	Yes	Yes (6)	Liver	NR	AWD (6)
30	20	15	M	Ileum	Localized	–	Yes	Yes (12)	Liver	NR	NR
31	21	36	F	Jejunum	NR	–	NR	NR	–	–	NR
32	22	49	F	Jejunum	Localized	–	Yes	No	–	–	NED (20)
33	2	25	F	Ileum	Metastatic	Liver	Yes	Yes (15 → 47)	Liver → ovary and peritoneal dissemination	Resection	AWD (47)
34	23	28	F	Small intestine	Localized	–	Yes	Yes (109)	Mesenteric LN	Resection	NED (161)
35	23	27	F	Small intestine	Metastatic	Lung and peritoneum	No	–	–	–	DOD (4)
36	23	33	M	Small intestine	Metastatic	Regional LN	Yes	Yes (2)	Mesenteric LN and Liver	None	DOD (8)
37	23	48	M	Small intestine	Localized	–	Yes	Yes (NR)	NR	NR	DOD (NR)
38	23	27	M	Small intestine	Metastatic	Peritoneum	Yes	No	–	–	NED (2)
39	24	47	F	Small intestine	Localized	Sigmoid colon direct invasion	Yes	Yes (1)	Multiple abdominal LN	NR	NR
40	25	12	M	Ileum	Localized	–	Yes	Yes (NR)	Mesenteric LN and multi liver	Resection and chemotherapy (no details)	AWD (18)
41	26	28	M	Ileum	Metastatic	Regional LN and liver	No	–	–	–	AWD (8)
42	27	29	M	Jejunum	Metastatic	Iliac LN	No	–	–	–	DOD (25)
43	PC	38	F	Jejunum	Metastatic	Regional LN	Yes	Yes (36)	Liver	Resection	NED (72)

*LN* lymph node, *AWD* alive with disease, *DOD* dead of disease, *NR* not reported, *NED* no evidence of disease, *PC* present case

## Conclusions

We report a patient with CCSLTGT with lymph node and liver metastases, who continues to survive 6 years after the initial surgical resection. It is important to follow patients with contrast CT every 3 months to ensure that radical resection of any recurrence can occur in a timely fashion.

## Data Availability

Data sharing is not applicable to this article, since datasets were neither generated nor analyzed for the case series.

## Abbreviations

CCSLTGT: Clear cell sarcoma-like tumor of the gastrointestinal tract
CT: Computed tomography
CCS: Clear cell sarcoma
EWSR1: Ewing sarcoma breakpoint region 1
GIST: Gastrointestinal stromal tumors
PET–CT: Positron emission tomography–CT
EOB-MRI: Gadoxetic acid-enhanced magnetic resonance imaging
CD56: The neural cell adhesion molecule
CAM 5.2: Anticytokeratin
CD45: Lymphocyte common antigen
ɑ-SMA: α-Smooth muscle actin
FISH: Fluorescence in situ hybridization
